# Clinical value of serum SIRT1 combined with uterine hemodynamics in predicting disease severity and fetal growth restriction in preeclampsia

**DOI:** 10.5937/jomb0-37645

**Published:** 2024-06-15

**Authors:** Tongjun Ge, JianYing Kong

**Affiliations:** 1 Qufu Normal University Hospital, Qufu City, China; 2 Qufu Peopležs Hospital, Department of Imaging, Qufu City, China

**Keywords:** preeclampsia, serum SIRT1, uterine hemodynamics, disease severity, fetal growth restriction, preeklampsija, serum SIRT1, hemodinamika materice, ozbiljnost bolesti, ograničenje rasta fetusa

## Abstract

**Background:**

To investigate the effect and correlation of serum SIRT1 combined with uterine hemodynamic parameters on disease severity and fetal uterine growth restriction in the progression of preeclampsia, and to evaluate its clinical value as potential markers.

**Methods:**

A total of 100 patients with preeclampsia who were hospitalized in Qufu Normal University Hospital from June 2017 to June 2021 were selected as the research objects. According to the severity, they were divided into Mild group (62 cases) and Severe group (38 cases), and according to whether the fetal growth restriction was combined or not, they were divided into the Combined fetal growth restriction group (56 cases) and the Uncomplicated fetal growth restriction group (44 cases). Serum SIRT1 levels and uterine artery hemodynamic parameters were detected, and spearman analysis was used to evaluate the association of serum SIRT1 levels and uterine artery hemodynamic parameters (peak-to-trough ratio of arterial blood velocity, pulsatility index, resistance index) with disease severity (systolic blood pressure, diastolic blood pressure, and random urinary protein levels) and fetal growth restriction (femoral length, biparietal diameter, head circumference and neonatal weight); unsupervised PCA analysis, supervised PLS-DA analysis, Cluster heat map analysis, ROC curve and AUC analysis were used to evaluate the diagnostic value of serum SIRT1 levels combined with uterine artery hemodynamic parameters in the severity of disease and fetal growth restriction in patients with preeclampsia.

**Results:**

Serum SIRT1 levels was decreased in patients with severe preeclampsia (p < 0.0001), arterial blood flow velocity peak-to-trough ratio, pulsatility index and resistance index were increased (p < 0.001; p < 0.0001), and serum SIRT1 levels and uterine artery hemodynamic parameters were closely related to disease severity (p < 0.001; p < 0.0001). In addition, the levels of serum SIRT1 in patients with preeclampsia combined with fetal growth restriction was decreased (p < 0.0001), the peak-to-trough ratio of arterial blood flow velocity, pulsatility index and resistance index were increased (p < 0.0001), and serum SIRT1 levels and uterine artery hemodynamics were closely related to fetal growth restriction (p < 0.0001). Unsupervised PCA analysis and supervised PLS-DA analysis showed that patients with different severity of disease and patients with or without fetal growth restriction were similar within groups, and there were significant differences between groups; cluster heat map analysis showed that mild and severe groups were stratified clustering, the combined fetal growth restriction group and the uncombined group were hierarchically clustered; ROC curve and AUC analysis showed that serum SIRT1 levels combined with uterine artery hemodynamic parameters had a significant effect on the severity of preeclampsia and whether combined with fetal growth restriction high diagnostic value.

**Conclusions:**

Serum SIRT1 combined with uterine hemodynamic parameters in preeclampsia is closely related to disease severity and fetal growth restriction, and is expected to become potential biomarkers for early clinical intervention in patients.

## Abstract (sr)

**Uvod:**

Cilj je bio da se ispita efekat i korelacija serumskog SIRT1 u kombinaciji sa hemodinamskim parametrima materice na težinu bolesti i restrikciju fetalnog rasta materice u progresiji preeklampsije, i da se proceni njihova klinička vrednost kao potencijalnih markera.

**Metode:**

Kao objekti istraživanja odabrano je ukupno 100 pacijenata sa preeklampsijom koji su bili hospitalizovani u Univerzitetskoj bolnici Kufu Normal od juna 2017. do juna 2021. godine. Po težini su podeljeni u blagu grupu (62 slučaja) i tešku grupu (38 slučajeva), a prema tome da li je ograničenje rasta fetusa kombinovano ili ne, podeljeni su u grupu kombinovanog ograničenja rasta fetusa (56 slučajeva) i grupa za ograničenje rasta fetusa bez komplikacija (44 slučaja). Detektovani su nivoi SIRT1 u serumu i hemodinamski parametri uteralne arterije, a Spirmanova analiza je korišćena za procenu povezanosti nivoa SIRT1 u serumu i hemodinamskih parametara uteralne arterije (odnos vrha do najniže vrednosti brzine arterijske krvi, indeksa pulsiranja, indeksa otpornosti) sa ozbiljnošću bolesti (sistolni krvni pritisak, dijastolni krvni pritisak i nasumični nivoi proteina u urinu) i ograničenje rasta femura (dužina femura, biparietalni prečnik, obim glave i neonatalna težina). Nenadzirana PCA analiza, nadgledana PLS-DA analiza, analiza klaster toplotne mape, ROC kriva i AUC analiza korišćene su za procenu dijagnostičke vrednosti nivoa SIRT1 u serumu u kombinaciji sa hemodinamskim parametrima uteralne arterije u težini bolesti i ograničenju rasta fetusa kod pacijenata sa preeklampsijom.

**Rezultati:**

Nivoi SIRT1 u serumu su smanjeni kod pacijenata sa teškom preeklampsijom (p < 0,0001), odnos brzine protoka arterijske krvi do najniže vrednosti, indeks pulsacije i indeks otpornosti su povećani (p < 0,001; p < 0,0001), a nivoi SIRT1 u serumu i materice hemodinamski parametri arterije bili su usko povezani sa težinom bolesti (p < 0,001; p < 0,0001). Pored toga, nivoi serumskog SIRT1 kod pacijenata sa preeklampsijom u kombinaciji sa ograničenjem rasta fetusa su smanjeni (p < 0,0001), povećan je odnos pik-na-niže brzine arterijskog krvotoka, indeks pulsacije i indeks otpornosti (p < 0,0001) , a nivoi SIRT1 u serumu i hemodinamika uteralne arterije bili su usko povezani sa ograničenjem rasta fetusa (p < 0,0001). Nenadzirana PCA analiza i nadgledana PLS-DA analiza su pokazale da su pacijenti sa različitom težinom bolesti i pacijenti sa ili bez ograničenja fetalnog rasta bili slični unutar grupa, i da su postojale značajne razlike izme|u grupa. Analiza klaster toplotne mape je pokazala da su blage i teške grupe bile stratifikovane u grupisanje, kombinovana grupa za ograničenje fetalnog rasta i nekombinovana grupa su hijerarhijski grupisane; ROC kriva i AUC analiza su pokazale da nivoi SIRT1 u serumu u kombinaciji sa hemodinamskim parametrima uteralne arterije imaju značajan uticaj na težinu preeklampsije i da li su kombinovani sa ograničenjem rasta fetusa visoku dijagnostičku vrednost.

**Zaključak:**

Serum SIRT1 u kombinaciji sa hemodinamskim parametrima materice kod preeklampsije je usko povezan sa težinom bolesti i ograničenjem rasta fetusa, i očekuje se da će postati potencijalni biomarkeri za ranu kliničku intervenciju kod pacijenata.

## Introduction

Preeclampsia (PE) refers to an idiopathic disease after 20 weeks of gestation, which occurs in a multi-stage and multi-step process, with clinical phenotypes such as increased blood pressure, abdominal pain, headache, nausea, and other symptoms [1] [2]. Symptoms such as renal failure, liver failure and cardiac arrest can be complicated, and in severe cases, the safety of the mother and baby can be endangered [1] [2]. In recent years, the incidence of preeclampsia has gradually increased. According to relevant data, the incidence of preeclampsia can reach 7% [1]. Fetal growth restriction (FGR) is mainly due to the low invasive ability of trophoblast cells and poor remodeling of the uterine spiral artery, which leads to placental ischemia and hypoxia, which affects fetal development [1] [2]. Preeclampsia combined with FGR is relatively common, the onset of gestational age is generally earlier, the functional development of fetal organs is immature, and the incidence of adverse pregnancy outcomes is high [1] [2]. The family brings great adverse effects, so the diagnosis and treatment of patients with preeclampsia complicated by fetal growth restriction has always been the focus of clinical attention.

Sirtuins are a class of nicotinamide adenine dinucleotide (NAD^+^)-dependent sirtuins [1]. Under physiological conditions, Sirtuins regulate the generation of reactive oxygen species by regulating the acylation/ deacylation process of antioxidant enzymes such as SOD1 and SOD2 [1] [2]. It can also regulate the activity of key metabolic enzymes such as mitochondrial respiratory chain complexes and the activation and transplantation of signaling pathways such as NF-κB and AKT through the same mechanism, thereby regulating the production of adenosine triphosphate and inflammatory factors, thereby participating in intracellular important physiological processes such as gene transcription, energy metabolism, and antioxidant defense are important for maintaining the stability of the intracellular environment and normal pregnancy [1]. When the levels of Sirtuins is down-regulated, their protective effect is also weakened, which can induce abnormalities such as maintenance stress, excessive inflammatory response, and energy metabolism disorders, leading to the occurrence of pathological pregnancy such as preeclampsia, GDM, and ICP [1]. In addition to being closely associated with the above-mentioned pathological pregnancies such as spontaneous preterm birth, preeclampsia, GDM, ICP, and fetal growth restriction, the Sirtuins family is also altered in other pathological pregnancies such as pregnancy depression and hyperemesis gravidarum. Among them, the sirtuin regulator 1-related enzyme (SIRT1) has been shown to play an important role in various pathophysiological processes [1] [2] [3]. In addition, during pregnancy, the uterine artery will undergo some physiological changes. After pregnancy, the blood vessels will gradually straighten and thicken, and the blood flow rate will increase, showing a state of low resistance and high flow [1]. The kinetic parameters will decrease accordingly. Studies have shown that uterine artery hemodynamic parameters in patients with preeclampsia are significantly higher than those in normal pregnant women [1]. Ultrasound technology enables non-invasive and real-time assessment of hemodynamic parameters in vital organs of pregnant women and fetuses [1]. However, to date, the roles of serum SIRT1 and uterine hemodynamics in the progression of preeclampsia are not fully understood. The aim of this study was to discover the effect and correlation of serum SIRT1 combined with uterine hemodynamic parameters on disease severity and fetal uterine growth restriction in the progression of preeclampsia, and to evaluate its clinical value as a potential marker.

## Materials and methods

### General information

A total of 100 patients with preeclampsia who were hospitalized in Qufu Normal University Hospital from June 2017 to June 2021 and underwent cesarean section on alternative days were selected. The clinical data of all subjects were collected, including age, body mass index, gestational age, number of gestations, number of deliveries, systolic blood pressure, diastolic blood pressure, and random urine protein detection. This study complied with the Declaration of Helsinki and the relevant regulations of clinical trial research in China. All subjects signed an informed consent form or authorized their family members to sign before enrollment. The study was approved by the ethics committee of the Qufu Normal University Hospital, and all sample collection met the requirements of quality management standards for clinical trial research.

### Inclusion and exclusion criteria

Inclusion criteria: (1) Meet the diagnostic criteria for preeclampsia in »Guidelines for the Diagnosis and Treatment of Hypertensive Disorders in Pregnancy (2020)«; (2) Patients and their families choose to undergo cesarean delivery; (3) All patients are singletons; (4) Age 24–40 years old; (5) Pregnancy times 3 times. Exclusion criteria: (1) those conceived by assisted reproductive technology; (2) those with hypertension before pregnancy; (3) severe kidney disease, chronic hypertension, autoimmune disease, liver disease; (4) gestational diabetes, placental abruption and other obstetric complications.

### Grading criteria for preeclampsia

The patients with preeclampsia were divided into mild group and severe group according to the severity of the disease. Professional doctors measured three consecutive measurements by electronic blood pressure, with systolic blood pressure (SBP) and diastolic blood pressure (DBP) defined as the average of the three measurements. Mild: systolic blood pressure greater than or equal to 140 mmHg, and/or diastolic blood pressure greater than or equal to 90 mmHg after 20 weeks of pregnancy; proteinuria greater than or equal to 0.3 g/24h, or random urine protein (+). Severe (if any of the following symptoms is present): pregnant women’s blood pressure continues to rise, systolic blood pressure is greater than or equal to 160 mmHg, and (or) diastolic blood pressure is greater than or equal to 100 mmHg; proteinuria is greater than or equal to 5 g/24h, or random urine protein (+++); elevated levels of liver enzymes alanine aminotransferase or aspartate aminotransferase, abnormal liver function; urine output less than 17 mL/h, or urine output less than 400 mL/24h, or serum creatinine greater than 106 μmol/L, abnormal renal function; anemia, intravascular hemolysis, jaundice, or platelets less than 1011/L; persistent upper abdominal pain, subcapsular hematoma, or liver rupture; persistent headache, visual disturbance, or other symptoms of cranial nerve injury; oligohydramnios, embryonic growth limited.

### Evaluation of fetal growth restriction

According to the criteria of »Expert Consensus on Fetal Growth Restriction (2019 Edition)«, patients with preeclampsia were divided into combined fetal growth restriction and non-fetal growth restriction. Four-dimensional color Doppler ultrasound was performed on pregnant women to record the femoral length, biparietal diameter and head circumference of the fetus; after the fetus was born, the neonatal weight was recorded, and the birth weight of the fetus was less than 2 standard deviations of the average weight for gestational age or less than the 10th percentile of normal weight for the same age.

## Observation indicators and methods

### Serum sample collection

Blood samples were collected within 24 hours of patient admission. A vacuum blood collection tube was used to collect 2 mL of cubital venous blood from the subjects, and the upper serum was collected by centrifugation. Set standards, samples to be tested and blank wells, refer to the steps in the kit instructions, and use the SIRT1 ELISA (EH427RB, 96 tests, Thermo Fisher Scientific Inc.) detection kit. The absorbance was detected by a microplate reader, and the standard curve was calculated from the absorbance of the standard to obtain the concentration of SIRT1 in serum samples.

### Measurement of uterine artery hemodynamic parameters

Pregnant women were kept in the supine or lateral position, breathing calmly, and GE Voluson E8 three-dimensional color Doppler ultrasound system (GE, USA) was used to explore the fetal structure, placenta and amniotic fluid. The ultrasound probe was placed in the groin, CDFI was started, the sampling frame was placed above the intersection of the uterine artery and the external iliac artery, and 1 cm away from the external iliac artery, the sampling volume was 2 mm, and the angle between the sampling line and the blood flow direction was <60°. Uterine artery blood flow parameters were measured, including arterial systolic peak flow velocity (S) and diastolic flow velocity (D). Time-averaged peak flow velocity (TAPV) was the time-averaged peak blood flow velocity in a cardiac cycle. Calculate the pulsatility index (PI) = (S-D)/TAPV; calculate the resistance index (RI) = (S-D)/S; calculate the peak-to-valley ratio of blood flow velocity = S/D.

### Statistical analysis

SPSS 22.0 statistical software was used to analyze and process the data, and GraphPad 8.0 software was used for drawing. Shapiro Wilk test was used to determine the normality of continuous variables. Statistical differences between the groups were assessed using Student T test or the Mann–Whitney U test for continuous variables. Correlation analyses were performed using Spearman’s test. Predictive validity of SIRT1 levels combined with uterine artery hemodynamic parameters for the severity of disease and fetal growth restriction in patients with preeclampsia was assessed using unsupervised PCA analysis, supervised PLS-DA analysis, Cluster heat map analysis, receiver operator curves (ROC) and corresponding results for the area under the curve (AUC). p <0.05 was considered statistically significant.

## Results

### Comparison of clinical data

The 100 patients with preeclampsia were graded according to the severity. There were 62 patients with mild preeclampsia and 38 patients with severe preeclampsia. The levels of systolic blood pressure (168.55±5.08) mmHg, diastolic blood pressure (118.13±9.73) mmHg and random protein (168.55±5.08) g/24h in the severe preeclampsia group were higher than those in the mild group (p < 0.0001) (Table 1). There were no significant differences in age, body weight, gestational age, number of pregnancies and number of deliveries between the two groups (Table 1).

**Table 1:** Comparison of clinical data of patients with preeclampsia grouped by severity.

	Mild Group<br>n=62	Severe Group<br>n=38	P-value
Age (year)	31.53±4.80	32.68±4.54	0.2376
BMI kg/m^2^	25.01±0.87	25.09±0.82	0.6211
Gestational Age<br>(week)	39.34±0.87	39.34±1.02	0.9859
Number of<br>Gestations (time)	1.74±0.77	1.92±0.82	0.2717
Number of<br>Deliveries (time)	1.53±0.67	1.68±0.70	0.2825
Systolic Blood<br>Pressure (mmHg)	149.32±5.84	168.55±5.08	<0.0001
Diastolic Blood<br>Pressure (mmHg)	94.56±2.51	118.13±9.73	<0.0001
Random Urine<br>Protein Detection<br>(g/24h)	2.74±1.01	6.39±0.91	<0.0001

In addition, according to whether the 100 patients with preeclampsia were complicated with fetal growth restriction, there were 56 cases with fetal growth restriction and 44 cases without fetal growth restriction. Femoral length, biparietal diameter, head circumference and neonatal weight in the combined fetal growth restriction group were lower than those in the uncomplicated fetal growth restriction group (p < 0.0001) (Table 2). There were no significant differences in age, body weight, gestational age, number of pregnancies and number of deliveries between the two groups (Table 2).

**Table 2:** Comparison of clinical data of preeclampsia patients grouped according to whether they were combined with fetal growth restriction.

	Combined<br>FGR Group<br>n=56	Uncomplicated<br>FGR Group<br>n=44	P-value
Age (year)	31.39±4.56	32.70±4.85	0.1685
BMI (kg/m^2^)	25.01±0.94	25.08±0.71	0.6881
Gestational<br>Age (week)	39.36±1.00	39.32±0.83	0.8354
Number of<br>Gestations (time)	1.88±0.81	1.73±0.76	0.3544
Number of<br>Deliveries (time)	1.55±0.66	1.64±0.72	0.5501
Femoral length<br>(cm)	6.63±0.30	7.41±0.35	0.0001
Biparietal<br>Diameter (cm)	8.51±0.24	9.16±0.38	0.0001
Head<br>Circumference<br>(cm)	29.21±1.16	31.94±1.50	0.0001
Neonatal Weight<br>(kg)	2.55±0.12	2.82±0.09	0.0001

### Comparison of serum SIRT1 levels and uterine artery hemodynamics

**Figure 1:**
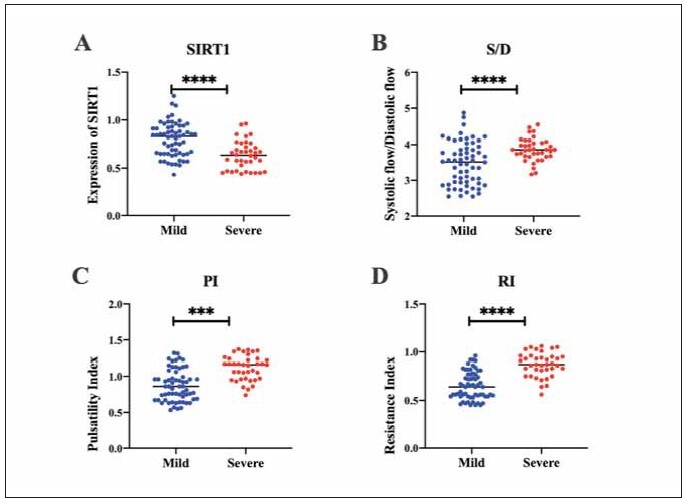
Serum SIRT1 levels and uterine artery hemodynamic parameters in patients with different severities of preeclampsia.<br>(A) Serum SIRT1 levels; (B) Peak-to-trough ratio of blood flow velocity; (C) Levels of pulsatility index; (D) Levels of resistance index. ***p<0.001; ****p<0.0001.

**Figure 2:**
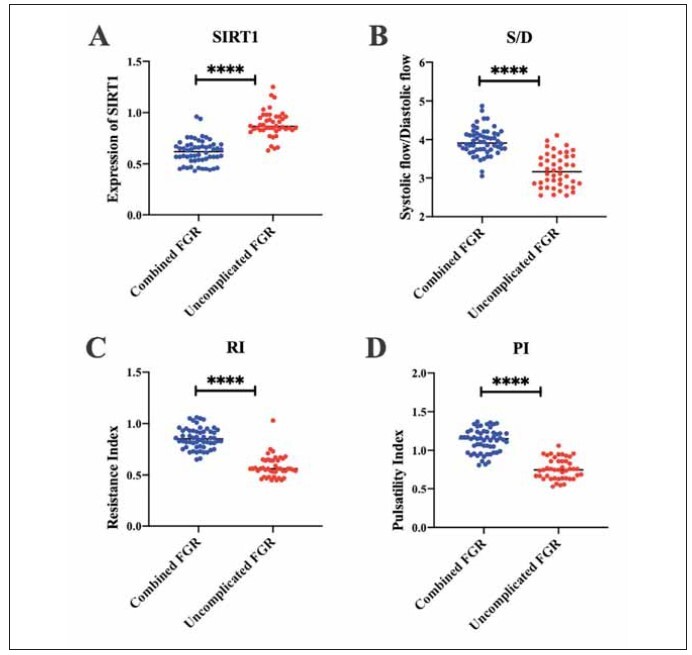
Whether serum SIRT1 levels and uterine artery hemodynamic parameters are combined with the levels in patients with or without fetal growth restriction and preeclampsia. (A) Serum SIRT1 levels; (B) Peak-to-trough ratio of blood flow velocity; (C) Levels of pulsatility index; (D) Levels of resistance index. ****p<0.0001.

We detected serum SIRT1 levels in all patients. The results showed that the levels of serum SIRT1 in patients with severe preeclampsia decreased, compared with the mild group (p < 0.0001) (Figure 1A); the peak-to-trough ratio of blood flow velocity, pulsatility index and resistance index were all increased in patients with severe preeclampsia, compared with the mild group (p < 0.001; p < 0.0001) (Figure 1B-D). In addition, the levels of serum SIRT1 was decreased in patients with preeclampsia combined with fetal growth restriction, compared with the combined group (p < 0.0001) (Figure 2A); the peak-to-trough ratio of arterial blood flow velocity, pulsatility index and resistance index were all increased in patients with preeclampsia combined with fetal growth restriction, compared with the uncombined group (p < 0.0001) (Figure 2B-D). These results suggest that serum SIRT1 levels and uterine artery hemodynamic parameters may be closely related to the severity of preeclampsia and the presence of fetal growth restriction.

### Correlation analysis of serum SIRT1 levels and uterine artery hemodynamics with disease severity

**Figure 3:**
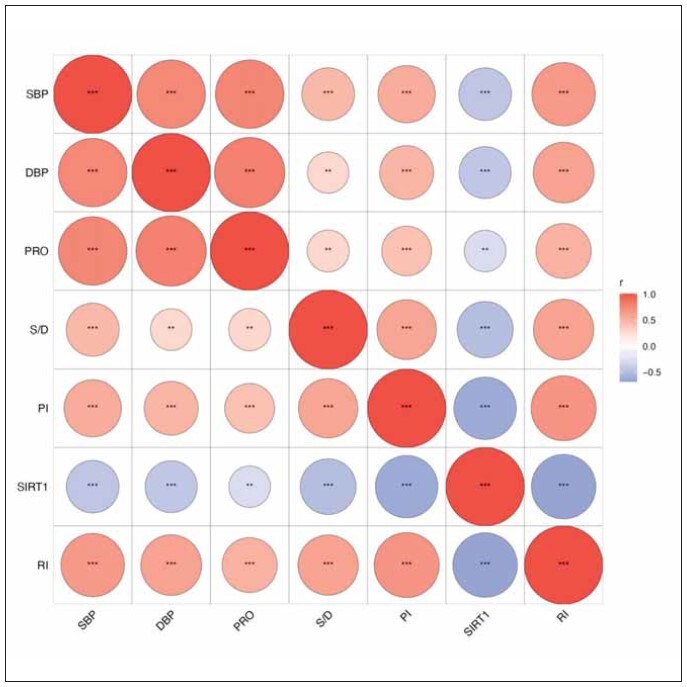
Correlation analysis of serum SIRT1 levels and uterine artery hemodynamics with the severity of preeclampsia. **p<0.01; ***p<0.001.

To examine the relationship between serum SIRT1 levels and uterine artery hemodynamics and disease severity, spearman correlation analysis was further performed, and the results showed that serum SIRT1 levels was negatively correlated with systolic blood pressure, diastolic blood pressure, and random urinary protein levels (p 0.001; p 0.01) (Figure 3); arterial blood flow velocity peak-to-trough ratio, pulsatility index, resistance index were positively correlated with systolic blood pressure, diastolic blood pressure and random urinary protein levels (p < 0.001; p < 0.01) (Figure 3). These results suggest that serum SIRT1 levels and uterine artery hemodynamics are closely related to the severity of preeclampsia.

### Correlation analysis of serum SIRT1 levels and uterine artery hemodynamics with fetal growth restriction

**Figure 4:**
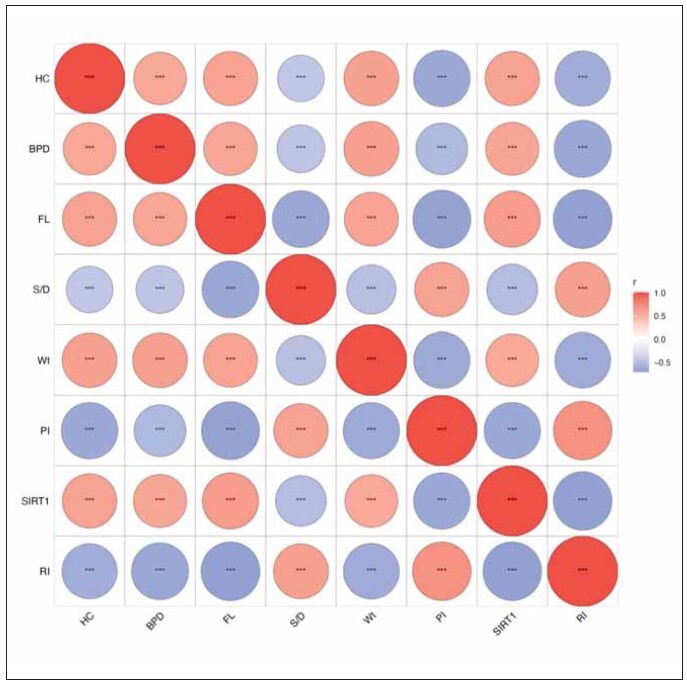
Correlation analysis of serum SIRT1 levels and uterine artery hemodynamics with preeclampsia complicated with fetal growth restriction. ***p<0.001.

To examine the relationship between serum SIRT1 levels and uterine artery hemodynamics and fetal growth restriction, spearman correlation analysis was further performed, and the results showed that serum SIRT1 levels was positively correlated with femoral length, biparietal diameter, head circumference and neonatal weight relationship (p < 0.001) (Figure 4); arterial blood flow velocity peak-to-trough ratio, pulsatility index, resistance index were negatively correlated with femoral length, biparietal diameter, head circumference, and neonatal weight (p < 0.001) (Figure 4). These results suggest that serum SIRT1 levels and uterine artery hemodynamics are closely related to preeclampsia complicated by fetal growth restriction.

### The diagnostic value of serum SIRT1 levels combined with uterine artery hemodynamics in predicting disease severity

**Figure 5:**
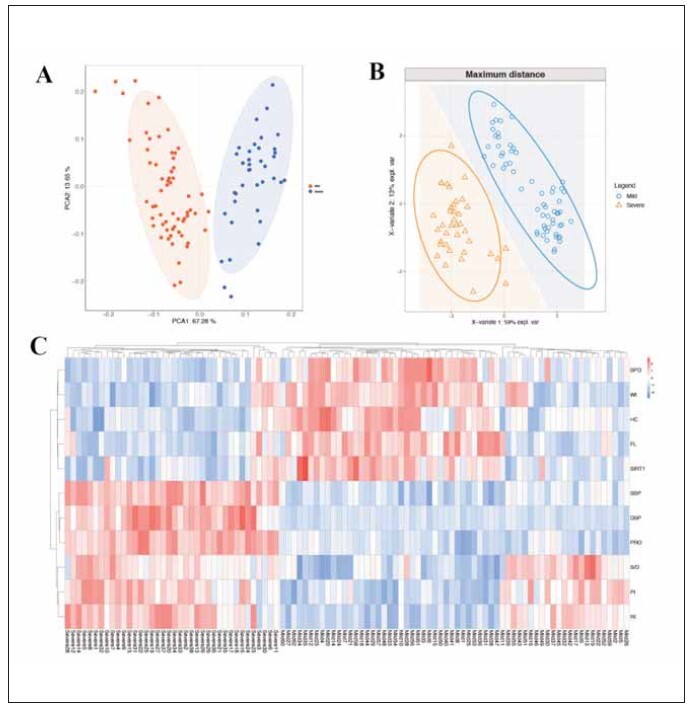
Discriminant analysis of serum SIRT1 levels combined with uterine artery hemodynamic parameters on the severity of preeclampsia. (A) PCA analysis of serum SIRT1 levels combined with uterine artery hemodynamic parameters in patients with different severity of preeclampsia; (B) PLS-DA analysis of serum SIRT1 levels combined with uterine artery hemodynamic parameters in patients with different severity of preeclampsia; (C) Between-group clustering heat map of serum SIRT1 levels combined with uterine artery hemodynamic parameters in patients with different severity of preeclampsia.

Unsupervised PCA analysis of the severity of preeclampsia with serum SIRT1 levels combined with uterine arterial hemodynamics showed that groups of different severity tended to be clustered and tended to be discrete (Figure 5A). The PLS-DA analysis also showed that the mild group and the severe group were significantly separated in the PC1 dimension, suggesting that the patients with different severity of preeclampsia were similar within the group and significantly different between the groups (Figure 5B). The heat map of cluster analysis showed that the mild group and the severe group were hierarchically clustered, suggesting that serum SIRT1 levels combined with uterine artery hemodynamic parameters could discriminate disease severity in patients with preeclampsia (Figure 5C).

**Figure 6:**
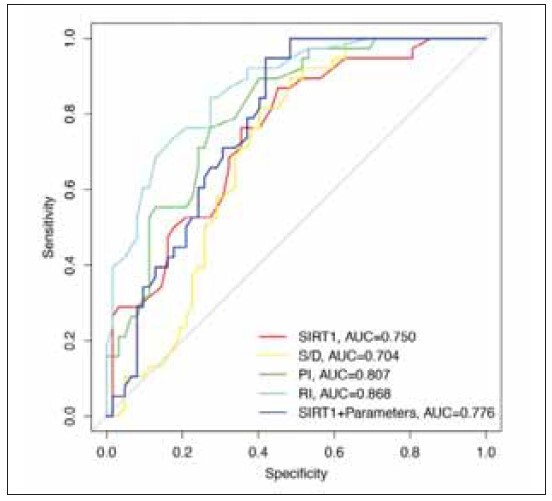
The diagnostic value of serum SIRT1 levels combined with uterine artery hemodynamic parameters for disease severity in patients with preeclampsia.

The diagnostic value of serum SIRT1 levels combined with uterine artery hemodynamic parameters in the diagnosis of disease severity in patients with preeclampsia was analyzed by ROC curve and AUC. The results showed that the AUC value of serum SIRT1 levels combined with uterine artery hemodynamic parameters (AUC=0.776) was higher than that of serum SIRT1 levels (AUC=0.750) and uterine artery hemodynamic flow velocity peak-to-trough ratio (AUC=0.704). (Figure 6), suggesting that serum SIRT1 levels combined with uterine artery hemodynamic parameters has a high diagnostic value for the severity of preeclampsia.

### The diagnostic value of serum SIRT1 levels combined with uterine artery hemodynamics in predicting fetal growth restriction

**Figure 7:**
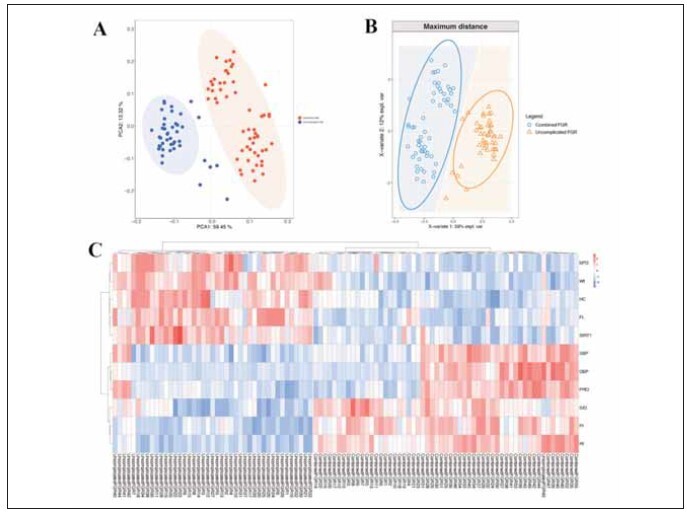
Discriminant analysis of serum SIRT1 levels combined with uterine artery hemodynamic parameters in preeclampsia complicated with fetal growth restriction. (A) PCA analysis of serum SIRT1 levels combined with uterine artery hemodynamic parameters; (B) PLS-DA analysis of serum SIRT1 levels combined with uterine artery hemodynamic parameters; (C) Between-group clustering heat map of serum SIRT1 levels combined with uterine artery hemodynamic parameters.

Unsupervised PCA analysis of fetal growth restriction in preeclampsia with serum SIRT1 levels combined with uterine artery hemodynamics showed that the groups tended to be clustered and tended to be discrete (Figure 7A); The PLS-DA analysis also showed that the group with fetal growth restriction and the uncombined group were significantly separated in the PC1 dimension, suggesting that the preeclampsia patients with or without fetal growth restriction were similar within the group, and the difference between the groups was significant (Figure 7B). Cluster analysis heat map showed that the combined and uncombined groups were hierarchically clustered, suggesting that serum SIRT1 levels combined with uterine artery hemodynamic parameters could differentiate fetal growth restriction in patients with preeclampsia (Figure 7C).

**Figure 8:**
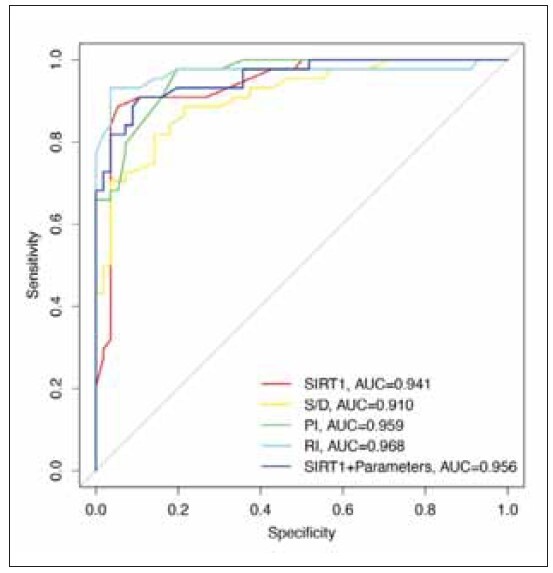
The diagnostic value of serum SIRT1 levels combined with uterine artery hemodynamic parameters in patients with preeclampsia complicated by fetal growth restriction.

The diagnostic value of serum SIRT1 levels combined with uterine artery hemodynamic parameters for fetal growth restriction in patients with preeclampsia was analyzed by ROC curve and AUC. The results showed that the AUC value of serum SIRT1 levels combined with uterine artery hemodynamic parameters (AUC=0.956) was higher than that of serum SIRT1 levels (AUC=0.941) and uterine artery hemodynamic flow velocity peak-to-trough ratio (AUC=0.910). (Figure 8), suggesting that serum SIRT1 levels combined with uterine artery hemodynamic parameters has a high diagnostic value for fetal growth restriction in preeclampsia.

## Discussion

In early pregnancy, trophoblasts invade the uterine spiral artery, remodel the uterine spiral artery, ensure the placenta engineering, and provide nutrients for the fetus [1]. Preeclampsia pregnant women have insufficient trophoblast invasion in early pregnancy, which leads to the disorder of uterine spiral artery remodeling and abnormal placenta formation, which lays a hidden danger for the occurrence and development of preeclampsia; the placental blood perfusion is insufficient, and placental ischemia and hypoxia will release cells Toxic substances enter the blood, further causing oxidative stress and vascular endothelial injury [1]. When the condition worsens, the uterine spiral artery rapidly atheroscleres, and the placental perfusion is further reduced and induces thrombosis and placental infarction. The severity of preeclampsia is associated with maternal and neonatal morbidity and thus mortality. Untreated, pre - eclampsia may have serious complications, such as eclampsia, liver rupture, stroke, pulmonary edema or renal failure, which are all fatal complications and are also associated with fetal prognosis, placental abruption, fetal growth increased risk of restriction, preterm birth, and stillbirth. Whether spontaneous or iatrogenic, neonates with preeclampsia may develop bronchopulmonary dysplasia and cerebral palsy. Preeclampsia may reduce the quality of health of the newborn and increase the risk of postpartum depression. In addition, preeclampsia and fetal growth restriction share common pathophysiological features, both of which are placental diseases [1]. Preeclampsia is also an independent risk factor for fetal growth restriction, and fetal growth restriction is a serious complication of preeclampsia [1]. At present, the pathogenesis of preeclampsia is still unclear, and specific clinical indicators are still lacking to predict the severity of preeclampsia and fetal growth restriction. Therefore, theoretically, if multiple disease- related biological indicators are combined, the sensitivity and specificity of prediction can be improved to a certain extent [1] [2]. We explored the clinical value of serum SIRT1 combined with uterine hemodynamics in preeclampsia in predicting disease severity and fetal growth restriction.

Trophoblast invasion is regulated by various pathways and factors, among which SIRT1 is involved in regulating various biological processes [1] [2]. Studies have found that SIRT1 can negatively regulate the nuclear factor kappa B (NF-κB) signaling pathway involved in the inflammatory process by activating the extracellular regulated protease (ERK) pathway, inhibiting the validation response of human granulosa cells and oocytes, and exerting an anti-apoptotic effect [1]. Maternal SIRT1 deficiency results in defective offspring embryo development during mouse embryonic development (1). Our study found that patients with severe preeclampsia increased systolic blood pressure, diastolic blood pressure and random protein levels, decreased serum SIRT1 levels, and femoral length, biparietal diameter, head circumference, and neonatal weight in patients with preeclampsia complicated by fetal growth restriction and serum SIRT1 levels decreased, suggesting that serum SIRT1 levels may be closely related to the severity of preeclampsia and whether combined with fetal growth restriction. In a study, the activity and expression of SIRT1 were significantly downregulated in aortic endothelial cells and smooth muscle cells of hypertensive model animals induced by Klotho haploid deficiency (KL+/-) [3] [4] [5] [6] [7] [8] [9] [10] [11] [12] [13] [14] [15] [16] [17] [18] [19] [20] [21] [22] [23] [24] [25] [26] [27] [28] [29] [30] [31] [32] [33]. We further conducted spearman correlation analysis and found that serum SIRT1 levels was negatively correlated with systolic blood pressure, diastolic blood pressure and random urine protein content, confirming that we previous assumptions. Moreover, we observed a positive correlation between SIRT1 levels and femur length, biparietal diameter, head circumference and neonatal weight.

In addition, ultrasound Doppler analysis can detect evidence of impaired uterine artery blood flow and placental perfusion before clinical manifestations of preeclampsia appear, and has become an effective method to predict adverse pregnancy outcomes such as preeclampsia [1]. Uterine artery blood flow can reflect the hemodynamic status of the mother. Under normal circumstances, in order to ensure the blood supply of the uterus and placenta, the uterine artery blood flow resistance will gradually decrease from the beginning of pregnancy, but the spiral artery remodeling in patients with preeclampsia adversely, the uteroplacental circulation resistance increases, which in turn causes the uterine artery resistance to increase [1]. Our study found that the peak-to-trough ratio of arterial blood flow velocity, pulsatility index and resistance index were increased in patients with severe preeclampsia, and the peak-to-trough ratio of arterial blood flow velocity, pulsatility index and resistance index in patients with preeclampsia complicated with fetal growth restriction Both were elevated, suggesting that uterine artery hemodynamic parameters may also be closely related to the severity of preeclampsia and the presence of fetal growth restriction. We further conducted spearman correlation analysis and found that the peak-to-trough ratio of arterial blood flow velocity, pulsatility index, and resistance index were positively correlated with systolic blood pressure, diastolic blood pressure, and random urine protein content, and were positively correlated with femur length, biparietal diameter, head circumference, neonatal body weights were negatively correlated, which also confirmed our earlier hypothesis.

Although no clinical studies have shown that the level or expression of SIRT1 is directly related to uterine artery hemodynamics, an in vitro study showed that the shear stress exerted by blood flow is related to the activity and expression of SIRT1. Therefore, we hypothesized that serum SIRT1 levels are associated with uterine artery hemodynamics [34]. Furthermore, to evaluate the diagnostic and predictive value of serum SIRT1 levels combined with uterine artery hemodynamics, we performed unsupervised PCA analysis, supervised PLS-DA analysis, and cluster heat map analysis, and found that serum SIRT1 levels combined with uterine artery hemodynamics The biological parameters can effectively distinguish between the disease severity and the complicated fetal growth restriction in patients with preeclampsia. Finally, the diagnostic value of serum SIRT1 levels combined with uterine artery hemodynamic parameters was further analyzed by ROC curve and AUC high diagnostic value. We found that serum SIRT1 levels combined with uterine artery hemodynamic parameters has a higher diagnostic value for the severity of preeclampsia compared to the levels of serum SIRT1 or uterine artery hemodynamic parameters.

## Conclusion

Serum SIRT1 combined with uterine hemodynamic parameters in preeclampsia is closely related to disease severity and fetal growth restriction, and is expected to become potential biomarkers for early clinical intervention in patients.

## Dodatak

### Acknowledgments

Not applicable.

### Funding

Not applicable.

### Conflict of interest statement

All the authors declare that they have no conflict of interest in this work.
